# Suitability of Optical, Physical and Chemical Measurements for Detection of Changes in Bacterial Drinking Water Quality

**DOI:** 10.3390/ijerph10115349

**Published:** 2013-10-25

**Authors:** Jenni Ikonen, Tarja Pitkänen, Ilkka T. Miettinen

**Affiliations:** Water and Health Unit, Department of Environmental Health, National Institute for Health and Welfare, P.O. Box 95, FI-70701 Kuopio, Finland; E-Mails: tarja.pitkanen@thl.fi (T.P.); ilkka.miettinen@thl.fi (I.T.M.)

**Keywords:** drinking water, bacterial contamination, parameter, water quality

## Abstract

In this study, different optical, physical and chemical measurements were tested for their capacity to detect changes in water quality. The tests included UV-absorbance at 254 nm, absorbance at 420 nm, turbidity, particle counting, temperature, pH, electric conductivity (EC), free chlorine concentration and ATP concentration measurements. Special emphasis was given to investigating the potential for measurement tools to detect changes in bacterial concentrations in drinking water. Bacterial colony counts (CFU) and total bacterial cell counts (TBC) were used as reference methods for assessing the bacterial water quality. The study consists of a series of laboratory scale experiments: monitoring of regrowth of *Pseudomonas fluorescens*, estimation of the detection limits for optical measurements using *Escherichia coli* dilutions, verification of the relationships by analysing grab water samples from various distribution systems and utilisation of the measurements in the case of an accidentally contaminated distribution network. We found significant correlations between the tested measurements and the bacterial water quality. As the bacterial contamination of water often co-occurs with the intrusion of matrixes containing mainly non-bacterial components, the tested measurement tools can be considered to have the potential to rapidly detect any major changes in drinking water quality.

## 1. Introduction

Prevention and control of unfavourable changes in drinking water quality are daily challenges in waterworks worldwide. The origin of raw water, water treatment techniques, and bacterial regrowth (biofilm formation) in distribution systems influences water quality and distribution systems can be considered as biological and chemical reactors that interact with transported water [1,2,3] . The growth potential of heterotrophic microbes in water distribution systems has been considered to have a relationship with the assimilable organic carbon (AOC) concentration in water [4] and in boreal regions, bacterial growth is known to be regulated by the availability of phosphorus [5]. Thus, efficient removal of nutrients is required to prevent bacterial regrowth in distribution systems. The quality of the distributed drinking water is also threatened by changes in water hydraulics and intrusions, *i.e.*, changes in water velocities, due to maintenance work such as pipe flushing, or injuries or other accidental events in the distribution system such as breaks in pipes [6,7]. Also heavy rainfall events can affect drinking water quality [8]. The monitoring of drinking water quality focuses on verification of the water quality in the distribution system [9]. Continuous monitoring of water quality in distribution systems is rare in routine water quality verification procedures and therefore transient deterioration of water quality in the system might not been detected. 

Bacterial drinking water quality is usually measured as heterotrophic plate count (HPC), commonly by cultivation on YEA or R2A media [10,11]. The ability of HPC to predict the total bacterial load of the water sample has been criticised since it has been reported that HPC’s have been only 25.2% [12] or less [13] of the total bacterial counts. Another method for detecting bacteria in the drinking water is total bacterial counting, utilising fluorochromes and an epiflurecence microscope [14,15,16]. Total bacterial cell count detects all, but do not differentiate between viable, including viable but not culturable (VBNC), and non-viable cells [17]. The viability of the bacterial cells can be observed by measuring adenosine triphosphate (ATP) [18,19], since all viable cells contain ATP [20]. Recently, rapid and cultivation-independent approaches combining ATP measurement and flow cytometry have been developed for monitoring microbial activity in the drinking water [13,21,22], bringing new options for use of ATP as an early warning tool for monitoring drinking water quality.

In addition to the aforementioned applications, various optical, physical and chemical measurements may provide useful information on the characteristics of the water. The applications include optical measurements of UV-absorbance at 254 nm, absorbance at 420 nm, turbidity and particle counts. On-line monitoring applications for these parameters have been tested in karst springs [23,24], in drinking water treatment [25] and distribution networks [26,27]. As the use of optical measurements require resources, *i.e.*, investments in equipment, the potential for conventional physical water quality measurements, including temperature, pH and electric conductivity (EC), to detect rapid water quality changes are also worthy of investigation [24]. Moreover, measurements of free chlorine concentration may have important value in water quality characterization especially in terms of controlling bacterial regrowth by ensuring a sufficient amount of residual disinfectant in the drinking water distribution networks [28]. 

In this work, the capacity of selected optical, physical and chemical measurements to detect microbiological impurities in the water was studied. Many of these measurements are already in use on a daily basis at waterworks and therefore have the potential to be utilised more efficiently as early warning tools for detecting changes in water quality. Different approaches were used to observe how bacterial regrowth or emerging bacterial contamination could be detected, utilising non-bacterial measurements, *i.e.*, UV-absorbance at 254 nm, absorbance at 420 nm, turbidity, particle counting, temperature, pH, EC, free chlorine concentration and ATP concentration. The relationships between measured parameters and bacterial counts were sought by following *Pseudomonas fluorescens* regrowth in nutrient solution and by estimating the detection limits of the measurements according to different concentrations of *Escherichia coli* in spring water. In addition, the detected relationships were verified by analysing drinking water samples from different distribution systems and utilising the measurements for drinking water accidentally contaminated by sewage.

## 2. Material and Methods

### 2.1. Monitoring Growth of *Pseudomonas fluorescens*

The bacterial strain *Pseudomonas fluorescens* P17, biotype 7.2 (ATCC 49642) was used as a test strain in this study. A nutrient solution for *P. fluorescens* was prepared with commercial spring water and contained 1 mg/L acetate C/L and 1.5 g/L inorganic nutrient salt solution [29]. The solution was sterilized in an autoclave (120 °C) for a minimum of 15 min. After sterilizing, zero samples were taken and *P. fluorescens* bacteria inoculation was pipetted into duplicate nutrient solution flasks (1,500 mL). The flasks were incubated at room temperature (20.9–26.6 °C) and samples were taken every day for one week after the inoculation, apart from the first experiment, in which the bacterial growth was followed for two weeks. The colony counts (CFU) were measured on Reasoner`s 2 Agar (R2A) medium (Difco, Detroit, MI, USA) incubated at 22 ± 2 °C for 7 days [10]. Acridine orange direct counting (AODC) [14] using an Olympus BX51TF epifluorescence microscope (Olympus Co. Ltd., Tokyo, Japan) was used to analyse the total bacterial counts (TBC). The experiment was repeated nine times. 

During the experiment, several optical and physical parameters were measured from the samples. UV-absorbance at 254 nm and absorbance at 420 nm were measured using a spectrophotometer (UV 1601, Shimadzu Corporation, Kyoto, Japan). Turbidity (at 860 nm) was measured using a turbidity meter (Turb 555-IR WTW, Weilheim, Germany) and for total particle counts, a particle counter WaterViewer S/N 604-2 (Pamas GmbH, Rutesheim, Germany) was used with a sensor having a 1 µm particle size detection limit (max. 120,000 particles/mL). The particle counter separated the number of particles per millilitre into 8 size classes in the ranges: 1–1.5, 1.5–2, 2–4, 4–8, 8–15, 15–25, 25–50 µm and >50 μm. Temperature, pH and electric conductivity were measured using a pH-meter (340i WTW, Weilheim, Germany). The ATP concentrations were measured with an ATP Biomass Kit HS Prod. No. 266-311 (BioThema AB, Handen, Sweden) using a luminometer (BioOrbit, Turku, Finland). 

### 2.2. Monitoring *Escherichia coli* in Spring Water

*E. coli* (ATCC 8739) was cultured on Tryptone soya agar (TSA) and incubated overnight at 36 ± 2 °C. The bacterial mass was suspended into a test tube that contained 5 mL of spring water (Novelle, Turku, Finland). Series of ten-fold dilutions were made into end volumes of 100 mL of spring water. The *E. coli* counts were measured on Cromocult^©^ coliform agar (CC) medium (Merck KgaA, Darmstadt, Germany). 

UV-absorbance at 254 nm and turbidity were measured from the samples, as described in Section 2.1. Total particle counts were measured utilising a particle counter SVSS-C (Pamas GmbH) with a sensor SLS 25/25 with a 0.5 µm detection limit (max. 13,000 particles/mL) that detected particles in the size classes of 0.5–0.6, 0.6–0.7, 0.7–0.8, 0.8–0.9, 0.9–1, 1–1.1, 1.1–1.5, 1.5–2, 2–3, 3–4, 4–5, 5–7, 7–10 10–15, 15–20 and >20 µm/mL. The experiment was repeated three times. The detection limit was defined based on the standard deviation and linearity of the results over the range of *E. coli* concentrations.

### 2.3. Monitoring of Water Samples from Distribution Systems

Drinking water samples were analysed from the drinking water distribution systems of five towns with populations of *ca.* 9,400–92,600 inhabitants and one rural community with a population of *ca.* 7,500 in central Finland. Water samples were collected in three consecutive sampling rounds between May and December in 2007 with a total of 52 water samples being analysed. In three towns (A, B, C), the raw water source was groundwater. In town D, artificially produced groundwater was distributed. Town E distributed drinking water originating from a mixture of groundwater and treated surface water. In the rural community F, groundwater was used. Post-chlorination was used as the disinfection in four towns (A, C, D and E). There was no disinfection in town B. UV disinfection was used in the waterworks of rural community F. The selection criterion of sampling locations from the distribution system was any prior detected water quality deterioration. At the time of sampling, sodiumthiosulphate was used to neutralize active chlorine from the samples. Water samples were delivered to the laboratory in coolers and analysed within 24 h. 

The optical and physical parameters were measured as described in Section 2.1 exception for the particle counter used with the two latter sampling rounds, which was the PAMAS SVSS-C with sensor HCB-LD 50/50 having a 1µm detection limit (max. 24,000 particles/mL) and measured size classes of 1–1.5, 1.5–2, 2–2.5, 2.5–3, 3–3.5, 3.5–4, 4–4.5, 4.5–5, 5–5.5, 5.5–6, 6–6.5, 6.5–7, 7–7.5, 7.5–8 µm/mL. The concentration of free chlorine was measured using a photometer (Chlorometer 1000, Palintest, Gateshead, UK). The heterotrophic plate count (HPC) was analysed on R2A medium (Difco) at 22 ± 2 °C for 7 days [10] and on Yeast extract agar (YEA) at 22 °C (ISO 6222, 1999). Counts of coliform bacteria (including *E. coli*) and intestinal enterococci were analysed with a membrane filtration method utilising the mEndo Agar Les medium (SFS 3016, 2001) and the Slanetz & Bartley medium (ISO 7899-2, 2000), respectively. The TBC was counted as described in Section 2.1. The nutrient concentrations were analysed from the distribution water system of town D (number of samples, N = 14). Assimilable organic carbon (AOC) was analysed according to Miettinen *et al.* [29] and total organic carbon (TOC) with a high temperature combustion method (5000 TOC analyser, Shimadzu). Microbially available phosphorus (MAP) was analysed according to Lehtola *et al.* [30] and the standard method SFS-EN 1189 (1997) was used to analyse total phosphorus (total P).

### 2.4. The Case of an Accidentally Contaminated Distribution Network

During this work we had an opportunity to utilise the measurements with drinking water samples (N = 3) originating from an accidental sewage contamination episode. The contamination led to a serious waterborne outbreak in the Nokia municipality in December 2007. Turbidity, particle counts, temperature, pH and EC were measured as described in Section 2.3. The cause of the contamination was an illegal connection between a sewage pipe and the distribution system [31]. 

### 2.5. Statistical Analysis

Statistical analyses were performed using PASW Statistics 18 for Windows (SPSS Inc., Chicago, IL, USA). The relationship between optical, physical and chemical parameters and bacterial counts was assessed using Spearman’s rank correlation test.

## 3. Results

The number of samples, and median and range (min–max) of the bacterial numbers in the *Pseudomonas fluorescens* experiment (Section 3.1) and the *E. coli* experiment (Section 3.2) are presented in Table 1.

**Table 1:** Descriptive statistics of bacterial numbers (CFU/mL and cells/mL) in the *Pseudomonas fluorescens* regrowth experiment and the *E. coli* experiments in spring water.

	N	Median	Min–Max
***Pseudomonas fluorescens***			
**colony counts**	74	9.3 × 10^5^	1.0 × 10^2^–8.8 × 10^6^
**total cell counts**	58	8.9 × 10^5^	1.9 ×10^2^–4.6 × 10^7^
***E. coli* colony counts**	20	3.05 × 10^4^	0–3.6 × 10^7^

### 3.1. Growth of *Pseudomonas fluorescens*

**Figure 1:**
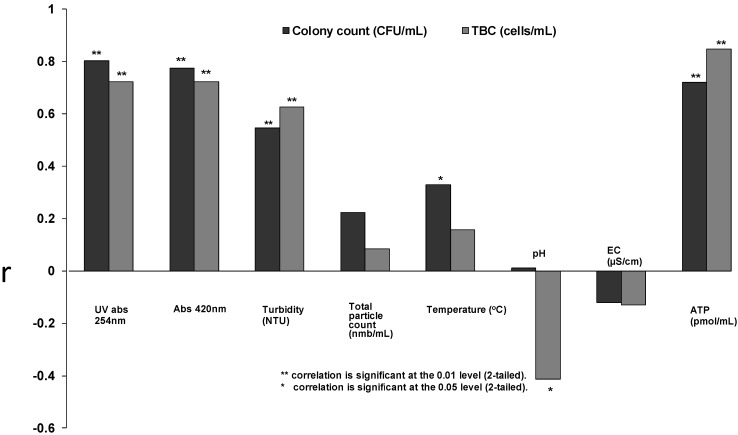
Spearmann’s rank correlation coefficients (r) of bacterial colony counts (CFU/mL) and total cell counts (TBC) with UV-absorbance at 254 nm (N = 77) and absorbance 420 nm (N = 82), turbidity (N = 64), total particle count (N = 47), temperature (N = 61), pH (N = 62), electric conductivity (N = 62) and ATP concentration (N = 33) in the *P. fluorescens* growth experiments in nutrient solution.

The mean *P. fluorescens* count (±SD) in the nutrient solution immediately after inoculation was 180 ± 80 CFU/mL. The stationary state of colony counts (3.3 × 10^7^ ± 1.1 × 10^6^ CFU/mL) and total cell counts (4.6 × 10^7^ ± 5.6 × 10^6^ cells/mL) was achieved before the fifth day of incubation at room temperature. Correlation coefficients between *P. fluorescens* colony counts and optical measurements showed an association with UV-absorbance at 254 nm (r = 0.802, *p* < 0.001) and absorbance at 420 nm (r = 0.774, *p* < 0.001), while turbidity showed a moderate relationship with *P. fluorescens* counts (r = 0.546, *p* < 0.001) (Figure 1). No relationship between particle and colony counts was detected. Instead, a connection between ATP concentration and the colony counts was found (r = 0.721, *p* < 0.001). Temperature (16.8–25.8 °C), pH (6.3–7.9) and EC (50–70 µS/cm) remained almost stable during the experiments. When the results of the measurements were compared with TBC, somewhat similar relationships were found (Figure 1). TBC and UV-absorbance at 254 nm and absorbance at 420 nm both exhibited significant correlations (r = 0.723, *p* < 0.001) while the relationship between turbidity and TBC was also significant (r = 0.626, *p* < 0.001). The highest correlation was found between ATP concentration and TBC (r = 0.847, *p* < 0.001). Colony counts and TBC correlated at the 99% significance level (r = 0.671, *p* < 0.001).

### 3.2. Detection of E. coli in Spring Water

**Figure 2:**
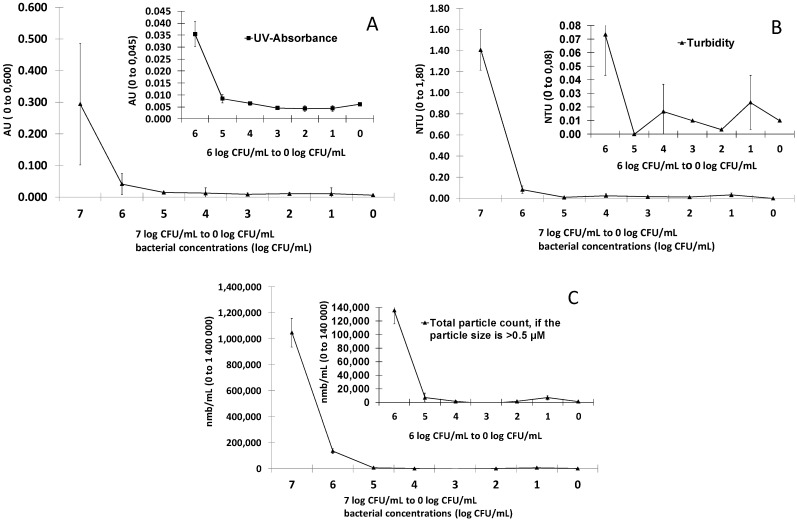
UV-absorbance at 254 nm (**A**), turbidity (**B**) and total particle count (**C**) values measured from samples with different *E. coli* concentrations (log CFU/mL). The measurements were repeated three times and error bars indicate the standard errors of the measurements.

Dilutions of the *E. coli* bacteria in spring water were prepared and optical parameters were measured. It was observed that UV-absorbance at 254 nm, turbidity and particle counts decreased over 85% when the bacterial counts decreased from 10^7^ CFU/mL to 10^6^ CFU/mL (Figure 2). The measured values of UV-absorbance at 254 nm and turbidity in the samples with an *E. coli* concentration of 10^6^ CFU/mL were 0.04 ± 0.01 absorbance units and 0.07 ± 0.03 NTU, respectively. When *E. coli* concentrations were below 10^6^ CFU/mL, the absorbance and turbidity values lost their relation to the bacterial counts and the measurements were evaluated to be non-reliable (Figure 2). In particle counting with the 0.5 µm sensor, a threshold value of 135,550 ± 19,839 particles/mL was achieved at the *E. coli* concentration of 10^6^ CFU/mL. With 10^5^ CFU/mL the threshold was 6,707 ± 6,557 particles/mL. In total, 97% of the counted particles were classified to a particle size fraction of 0.5–0.6 µm in accordance with the known size of *E. coli* cells (0.3–1.0 µm × 1.0–6.0 µm; Brenner and Staley [32]). The UV-absorbance, turbidity and particle count of the commercial spring water without *E. coli* addition was 0.006 absorbance units, 0.01 NTU and 1,100 particles/mL, respectively.

### 3.3. Monitoring of the Distribution System Water Samples

The descriptive statistics of the measured parameters from the analysed distribution water samples originating from six different distribution systems are presented in Table 2. One colony of coliform bacteria/1,000 mL was detected in three out of 34 drinking water distribution water samples. It was detected from town D, indicating the inefficiency of post-chlorination to prevent regrowth in this distribution. No *E. coli* or intestinal enterococci were found from the samples. Mean AOC, MAP, TOC and total phosphorus concentrations (±SD) in the distribution system of town D were 126 µg ± 30 AOC-C/L µg/L, 0.04 ± 0.05 µg/L, 2.7 ± 0.6 mg/L, and 1.7 ± 0.6 µg/L, respectively. 

**Table 2:** Descriptive statistics of the parameters measured from the drinking water distribution networks of five towns and one rural community in Finland.

	N	Median	Min–Max
**Optical**			
**Abs 420**	52	0.01	0.001–0.330
**UV Abs 254**	52	0.133	0.019–0.416
**Turbidity (NTU)**	52	0.257	0.04–27.4
**Total particle count (nmb/mL)**	51	1,718	1.1 × 10^2^–6.1 × 10^4^
**Physical**			
**Temperature (°C)**	52	7.7	5.1–21.0
**pH**	52	6.905	6.40–8.78
**EC(µS/cm)**	52	249	18–475
**Chemical**			
**Free chlorine (mg/L)**	18	0.225	0.10–0.64
**ATP (pmol/mL)**	52	41	0.04–119
**Bacterial**			
**HPC on R2A (CFU/mL)**	48	656	10–1.3 × 10^5^
**HPC on YEA (CFU/mL)**	51	3	0–5.3 × 10^3^
**TBC (cells/mL)**	52	55,784	1.1 × 10^3^–7.8 × 10^5^
**Coliforms (CFU/1,000 mL)**	34	BDL^1^	BDL^1^
**Enterococci (CFU/1,000 mL)**	34	BDL	BDL
**Nutrients**			
**AOC-C/L µg **	14	128	60.9–179.1
**TOC mg/L**	14	3	1.6–3.3
**MAP µg/L**	13	0.0085	0.0007–0.1533
**Total-P µg/L**	14	1.85	0.6–3.0

^1^ Below the detection limit.

**Figure 3:**
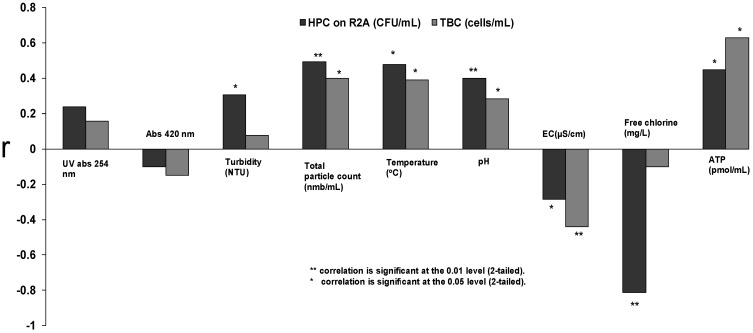
Spearmann’s rank correlation coefficients (r) between bacterial water quality (HPC on R2A and TBC) and optical, physical and chemical measurements in analysis of drinking water distribution system samples.

Figure 3 shows the relationships between bacterial counts (HPC on R2A and TBC) and measured non-bacterial parameters. With the distribution system samples, no relationship between absorbance measurements and bacterial counts were found and turbidity displayed only a slight connection with HPC on R2A (r = 0.305, *p* = 0.035). In contrast to the *P. fluorescens* experiments, particle counts exhibited a significant correlation with HPC on R2A when true distribution system samples were analysed (r = 0.493, *p* < 0.001) and also had a positive connection with TBC (r = 0.286, *p* < 0.004). Moreover, particle counts had a connection with HPC on YEA (r = 0.400, *p* = 0.044), (results that were observed with YEA are not shown). The temperature of water in the distribution systems had a positive connection with HPC on R2A (r = 0.478, *p* < 0.001), with HPC on YEA (r = 0.435, *p* < 0.001) as well as with TBC (r = 0.390, *p* = 0.004). For the TBC, a negative connection (r = −0.439, *p* < 0.001) was found with EC. pH exhibited a positive connection with HPC on R2A (r = 0.400, *p* < 0.005) and with TBC (r = 0.284, *p* < 0.042). Nonetheless, the best surrogate for HPC on R2A in distribution system water samples was free chlorine concentration, which exhibited the highest value for the correlation coefficient (r = −0.811, *p* < 0.001). Furthermore, the concentration of ATP had a positive connection with HPC on R2A (r = 0.449, *p* < 0.010) and with TBC (r = 0.629, *p* < 0.001). HPC on YEA had a significant positive connection with TOC (r = 0.815, *p* < 0.001) and a significant negative connection with total-P (r = −0.704, *p* = 0.002). 

### 3.4. The Case of an Accidentally Contaminated Distribution Network

The drinking water distribution system of Nokia town was heavily contaminated with a purified sewage intrusion, resulting in the exposure of inhabitants to a wide range of faecal microbes including norovirus, *Campylobacter*, *Giardia*, *Salmonella* as well as high concentrations of faecal indicator bacteria, *E. coli*, intestinal enterococci and *Clostridium perfringens* [31].

**Figure 4:**
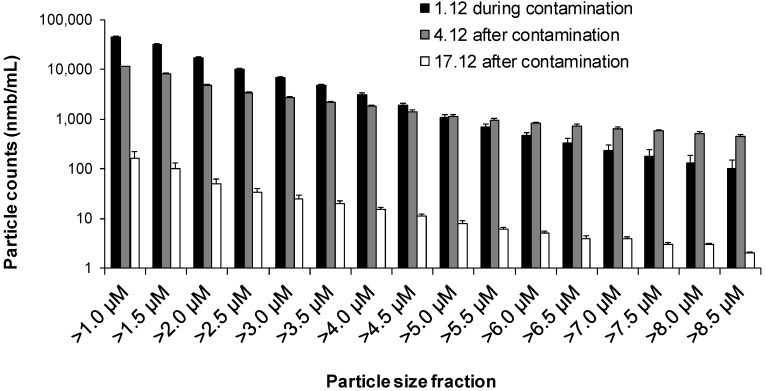
Particle size fractions during and after massive sewage contamination in Nokia municipality.

Particle measurement with a 1 µm detection limit for the particle size was used to examine the drinking water samples originating from the contaminated distribution system of the town. The comparison of total particle counts and particle fractions (Figure 4) during and after the contamination event highlighted the ability of particle counting to function as an early warning tool. HPC on R2A in the water sample taken on 1 December 2007, during the contamination, exceeded 5.0 × 10^6^ CFU/mL. Three days after the contamination event, on the 4th December 2007, the HPC value had declined to 1.8 × 10^2^ CFU/mL. At the same time, the particle counts decreased from 4.4 × 10^4^/mL to 1.2 × 10^4^/mL (Δ74%) and turbidity decreased from 4.6 to 1.2 NTU (Δ74%). At two weeks later, 17 December 2007, the particle count in the water sample was much lower, being 1.7 × 10^2^/mL (Δ99.6%) and the turbidity value was 0.3 NTU (Δ94%).

## 4. Discussion

The vulnerability of drinking water distribution systems is well known and the need for rapid, though still affordable detection methods for bacterial quality monitoring of drinking water is well recognized. Drinking water quality deteriorations can be divided into two groups: they can be long-term regrowth problems [4,33,34] or short-term transient intrusion events that might not be noticed at all. Biological instability of the water that causes regrowth of microbes is a stable phenomenon when occurring in a distribution system. Thus it is relatively easy to detect, but difficult to remove. In contrast, the intrusion of contaminating substances into the drinking water distribution is usually caused by a temporary failure that may be difficult to detect, but is often relatively easily fixed, once the cause is identified. Furthermore, seasonal events may reduce the drinking water treatment efficiency [8,35] and subsequently cause a long-lasting reduction of the distributed drinking water quality. In this study, several laboratory-scale measurements were observed to have potential to work as tools in the detection of water quality changes caused by regrowth or the faecal contamination of water.

The optical density of a liquid sample measured as absorbance (UV-absorbance at 254 nm and absorbance at 420 nm) was observed to have a relationship with colony counts and total bacterial count during a *P. fluorescens* growth experiment (Figure 1). However, the connection between absorbance and colony counts and absorbance and total bacterial count was absent when analysing drinking water samples (Figure 3). The reason for the absence of the connection could be that the analysed samples did not contain substances that absorbed light at the measured wavelengths. The other explanation might be that the bacterial concentration in the analysed drinking water samples remained below the detection limit of the absorbance method. When we studied the detection limits of the optical measurements (UV-absorbance, turbidity and particle counts; Figure 2) using *E. coli* dilutions in spring water, it was concluded that bacterial concentrations higher than 10^6^ CFU/mL can be measured reliably with the used equipment. However, we tested only three bottle series and 10-fold dilutions. By using smaller dilution steps it might be possible to achieve a detection limit between 10^5^ and 10^6^ CFU/mL. In fact, the measured particles indicated that a level of 10^5^ CFU/mL would be achievable with the method, but so far we could not confirm the observation since the standard error remained high in our experiments. Our results are in accordance with earlier results reported by Chavez, Jimenez and Maya [36], which concluded that the monitoring of particle counts is possible when the faecal coliform count is between 10^5^ and 10^8^ MPN/100 mL. 

Turbidity measurements have been used for monitoring total particle concentration in drinking water samples in distribution systems. Turbidity is known as an indirect measurement method that does not provide quantitative information about the water quality. The use of turbidity has been criticised as inadequate and not sufficient to ensure the microbiological safety of a water supply [37]. More recently, particle counting measurements have been used to provide information about water quality in distribution systems [26], which supplements the information from turbidity measurements. When measuring samples from the contaminated distribution system (Figure 4), we observed that the particle counter equipped with a sensor with a 1 µm detection limit for the particle size would be sensitive enough when detecting contamination caused by sewage intrusion. We used the same 1 µm sensor in the *P. fluorescens* experiments but in the *E. coli* experiments, a sensor with 0.5 µm detection limit for the particle size was used instead. In the *E. coli* experiment, nearly all bacterial cells were found from the particle size fraction of 0.5 µm–0.6 µm. As *P. fluorescens* cells are approximately the same size as *E. coli*, the use of a 1 µm sensor could explain why no relationship was found between bacterial and particle counts in the *P. fluorescens* regrowth experiment. The observation suggests that some other substances (such as humus, minerals and organic particles) than bacterial cells are responsible for the increase in particle counts within the analysed distribution water samples. 

The correlation of bacterial counts with particle counts measured using a 1 µm sensor (Figure 3) give an indication that changes in the drinking water quality are rarely simply attributable to a pure bacterial contamination. This interpretation is supported by the earlier findings of Pronk, Goldscheider and Zophi [24], as they found that an increase in finer particles (0.9 µm–1 µm) indicated faecal contamination of water. The Nokia incident is a good example of the complexity of contamination events [31]. The samples analysed in this study from the distribution system that was contaminated with sewage water gave us verification that the optical measurements, such as turbidity and particle counting, could be useful as rapid signals in drinking water contamination situations. In the future studies, potential of the use of flow cytometry, which is an advanced form of particle quantification and qualification [21,38,39] should be exploited.

Changes in water temperatures have an effect on the bacterial quality of the distribution system and temperature has been reported as one of the best predictors for the spatiotemporal occurrence of HPC in the distribution systems [40]. During the summer months temperatures are usually higher and seasonal problems with water quality have been observed [1,4]. In this study, the impact of temperature was examined in drinking water samples with three assays for bacterial counts (HPC on R2A, HPC on YEA and TBC) and the relationships were observed. Although it could be assumed that temperature can serve as a parameter predicting bacterial regrowth in a distribution system, the use of temperature alone is insufficient. The main factor regulating the microbial growth is availability of nutrients in drinking water [4,5,41] while the use of disinfection, the pH of the water, and the pipe material also play important roles [42,43]. The regrowth of microbes in distribution systems is difficult to prevent completely, but it can be controlled with residual disinfectant in the distribution [28]. In fact, previous studies have shown that one of the best parameters to predict biological contamination events in the distribution systems is the measurement of free chlorine [44]. In the present study, free chlorine was clearly the best parameter for predicting HPC in the studied distribution system water samples, whereas pH showed only a moderate positive correlation (Figure 3). It has also been postulated that pH and HPC have a positive correlation in water where chlorine is being used as a disinfectant [40,45]. The higher pH value is understood to decrease the efficiency of chlorine disinfection, with chlorination most effective at a pH value of 5 [46]. We found in the *P. fluorescens* growth test a negative connection between bacterial counts and pH. This connection might be explained by aerobic heterotrophic respiration and formation of CO_2_ in the bottle since *P. fluorescens* is an obligate aerobe. 

The use of ATP measurement coupled with cultivation-independent flow cytometry method has been proposed [13,21,39]. In this study, the previously detected relationship between the colony counts and ATP [18,19] was confirmed during the growth of *P. fluorescens* as a proof of the presence of viable micro-organisms in the sample. However, ATP had an even better correlation with TBC than with colony counts, the result possibly being attributable to the presence of VBNC cells. 

## 5. Conclusions

Results here indicated the usefulness of optical, physical and chemical measurements in detecting impurities in drinking water and the ability of these measurements to be used to detect changes when drinking water is undergoing deterioration.

The results confirmed that although turbidity is a good basic measurement for detecting changes in drinking water quality, the particle count gives more precise information. Particle counting was also found to work as a feasible indicator of bacterial counts in a real water contamination incident. Our results also indicated that absorbance measurements (254 nm, 420 nm) have potential to work at some extend as tools that could be used for detecting bacterial regrowth and intrusions in drinking water systems. 

Detection limits for the optical measurements of UV-absorbance, turbidity and particle counting were high when pure cultures of bacteria were tested (limits were met at *E. coli* concentrations of 10^6^ CFU/mL). The uncertainty of measurements increased when bacterial concentrations decreased below the detection limits. It seemed that limit was also there with the analysed non-contaminated drinking water samples were relationship was not found when bacterial counts were below the 10^6^ CFU/mL. 

Free chlorine concentration exhibited the strongest correlation with HPC when analysing distribution system water samples, while ATP measurement was found to be a useful tool for assessing the viability of the bacterial cells. 
